# Study protocol of a multicenter randomized controlled trial of mindfulness-based cognitive therapy and treatment as usual in bipolar disorder

**DOI:** 10.1186/s12888-019-2115-6

**Published:** 2019-04-30

**Authors:** I. Hanssen, M. J. Huijbers, M. W. H. Lochmann-van Bennekom, E. J. Regeer, A. W. M. M. Stevens, S. M. A. A. Evers, M. Wensing, R. W. Kupka, A. E. M. Speckens

**Affiliations:** 1 Department of Psychiatry, Centre for Mindfulness, Radboud University Medical Centre, Postbus 9101, 6500 HB Nijmegen, The Netherlands; 2 Donders Institute for Brain, Cognition and Behaviour, Radboud University, Nijmegen, The Netherlands; 3 Department of Mood Disorders, Pro Persona, Mental Health Care, Tarweweg 2, 6534 AM Nijmegen, The Netherlands; 4 Altrecht, Institute for Mental Health Care, Outpatient clinic for Bipolar Disorders, Nieuwe Houtenseweg 12, 3524 SH Utrecht, the Netherlands; 5 Dimence Mental Health, Center for Bipolar Disorders, Pikeursbaan 3, 7411 GT Deventer, The Netherlands; 6 Trimbos-instutuut, Postbus 725, 3500 AS Utrecht, the Netherlands; 7 Radboud University Medical Centre, Institute for Quality in Health Care, Postbus 9101, 6500 HB Nijmegen, the Netherlands; 8 Department of Psychiatry, Amsterdam UMC, Vrije Universiteit, Amsterdam Public Health Research Institute, Oldenaller 1, 1081 HJ Amsterdam, the Netherlands

**Keywords:** Mindfulness-based cognitive therapy, Bipolar disorder, Randomized controlled trial; study protocol

## Abstract

**Background:**

Despite multiple pharmacological interventions, many people with bipolar disorder (BD) experience substantial residual mood symptoms, even in the absence of severe mood episodes, which have a negative impact on the course of illness and quality of life. Limited data are available on how to optimize treatment for BD, especially for those who suffer from persistent and residual depressive symptoms. Preliminary evidence suggests Mindfulness-Based Cognitive Therapy (MBCT) as a psychological treatment option for BD. This study aims to investigate whether adding MBCT to treatment as usual (TAU) will result in symptomatic and functional improvements in adults with BD compared to TAU alone.

**Methods/design:**

This study is a prospective, evaluator blinded, multicenter, randomized controlled trial of MBCT + TAU and TAU alone in 160 adults with bipolar type I and type II. Assessments will be conducted at baseline (T0), mid-treatment (Tmid), and at 3 (T1), 6 (T2), 9 (T3), 12 (T4), and 15 (T5) months follow-up. Primary outcome is post-treatment severity of depressive symptoms (Inventory of Depressive Symptomatology- Clinician administered). Secondary outcomes are severity of (hypo) manic symptoms, anxiety, relapse rates, overall functioning, positive mental health, and cost-effectiveness. As possible mediators will be assessed rumination of negative affect, dampening and rumination of positive affect, mindfulness skills, and self-compassion.

**Discussion:**

This study will provide valuable insight into the (cost-)effectiveness of MBCT on clinician- and self-rated symptoms of BD, relapse rates, positive mental health, and overall functioning.

**Trial registration:**

NCT03507647. Registered 25th of April 2018.

## Background

Bipolar disorder (BD) is a disabling, chronic condition characterized by recurrent (hypo) manic, depressive, and/or mixed episodes, affecting approximately 1.2% of adult men and 1.4% of adult women in the Netherlands [1]. BD belongs to the leading causes of years lost due to disability, and is associated with considerable economic, occupational, and social burden [2–4]. Illness-related disability is mostly accounted for by depressive symptoms and episodes [5, 6]. Similar to individuals with major depression, people with BD demonstrate a tendency to ruminate in response to negative affect [7]. This appears to create a vicious cycle of ruminative thinking, decreased interest and motivation, and a loss of positive affect, resulting in an increase in depressive symptoms. Furthermore, people with BD show a tendency to ruminate in response to positive affect as well [8], which has been found to be correlated with increased (hypo) manic symptoms [9, 10]. Despite multiple pharmacological options, people with BD experience substantial residual mood symptoms about half the time. Approximately 60% of people with BD relapse in a full-blown mood episode within two years after recovering from a previous mood episode [6, 11–13]. These persistent mood symptoms affect the course of BD and quality of life negatively [14]. Therefore, the management of BD requires additional psychological interventions [15, 16]. However, most psychological interventions used in the treatment of BD, such as Cognitive Behavioral Therapy (CBT), are more effective for people with major depression or in people with BD who suffered less than 12 mood episodes [16–18]. Limited data are available on how to optimize treatment for the whole population of people with BD, especially for those who suffer from persistent and residual mood symptoms.

Mindfulness-based approaches have recently been adopted to the Dutch national guidelines of bipolar disorders, adopting Mindfulness-Based Cognitive Therapy (MBCT) as a psychological treatment option in order to prevent depressive relapse in people with BD [19]. MBCT integrates meditative practices with elements of cognitive therapy and aims toward people developing a capacity to be aware of (distressing) thoughts, feelings, and bodily sensations in a non-judgmental way [20, 21]. MBCT has been shown promising results in a wide range of psychiatric disorders [22], including major depression (e.g. [23, 24]), and a limited effect in anxiety disorders [25]. Even though MBCT was adopted as a treatment option to the Dutch national guidelines of bipolar disorders, evidence about its effectiveness in BD is still scarce. There are a number of small pilot studies showing either reductions in severity of depressive and/or anxiety symptoms [26–28], or no improvement in depressive symptoms [29]. To date, only one randomized controlled trial of MBCT for BD has been conducted in 95 participants [30]. This study of Perich et al. found significant improvements in anxiety symptoms, but not in depressive symptoms after MBCT over the course of a 12-month follow-up period. However, they included remitted adults with BD only, which may have limited the possible range of symptom reduction and the clinical representativeness of this study. Furthermore, the drop-out rates at 12-month follow-up were high, with almost 65% of participants not completing follow-up measures. The current trial will provide the first high-level evidence of the effectiveness of MBCT in addition to treatment as usual (TAU) compared to TAU alone in euthymic or depressed individuals with BD, by including follow-up assessments up to 12 months after completion of treatment and keeping close track of treatment adherence and occurrence of (serious) adverse reactions.

The primary aim of this study is to compare post-intervention depressive symptom severity after MBCT + TAU and TAU alone. The secondary aims are to investigate whether MBCT is effective in reducing long-term depressive symptoms, and post-treatment and long-term (hypo) manic and anxiety symptom severity, relapse rates, and increasing overall functioning and positive mental health. Previous research has shown that the relationship between mindfulness practice and improvement in psychological symptoms is mediated by mindfulness skills, self-compassion, and rumination [31]. Therefore, it is expected that improvements in severity of depressive symptoms is mediated by mindfulness skills, self-compassion, and rumination of negative affect, and dampening and rumination of positive affect. Moreover, since there is some evidence that certain psychological interventions become less effective for people with BD who experienced 12 or more mood episodes [18], the current trial will investigate whether MBCT will be more effective in people with BD with less than 12 mood episodes compared to people with BD who suffered 12 or more mood episodes. Finally, from a societal perspective, the current study will assess whether implementing MBCT + TAU is cost-effective compared to TAU alone. It is expected that adding MBCT to TAU will result in a reduction of medical and societal costs.

In conclusion, the current study will be the first randomized controlled trial of MBCT for BD in the Netherlands and to the best of our knowledge the second RCT overall, providing high-level evidence of the relative long-term effectiveness of MBCT + TAU versus TAU for adults with BD. It will be offered in a multi-centre setting, using several outcome measures, including cost-effectiveness. If (cost-)effective, MBCT might widen the array of evident psychological interventions for BD.

## Methods/design

### Study design

This study is a prospective, evaluator blinded, multicenter, randomized controlled trial of MBCT + TAU versus TAU alone. Assessments will be conducted at baseline (T0), mid-treatment (Tmid), and at 3 (T1), 6 (T2), 9 (T3), 12 (T4), and 15 (T5) months follow-up. Participants randomized to the TAU condition will be able to participate in a MBCT intervention after they completed the study (15 months). The study protocol has been approved by the ethical review board CMO Arnhem – Nijmegen and is registered under number NL63319.091.17.

### Setting

The MBCT interventions will be provided at the Radboud University Medical Centre in Nijmegen, and at five specialized outpatient clinics for bipolar disorders in the Netherlands, including Altrecht (Utrecht), Pro Persona (Arnhem/Nijmegen/Tiel/Ede); Dimence (Zwolle), PsyQ (Rotterdam) and GGZ Breburg (Tilburg/Breda).

### Study population

The study population will consist of adults (18 years or older) with BD. The following inclusion criteria will be applied: 1) a confirmed diagnosis of bipolar I or bipolar II disorder, according to the Diagnostic and Statistical Manual of Mental Disorders – 5th edition (DSM-5) [32] and confirmed by using the Structured Clinical Interview for DSM-IV Axis I Disorders (SCID-I) [33]; 2) having suffered at least two confirmed lifetime depressive episodes, either current or in (partial) remission at baseline; 3) having suffered at least one mood episode (either depressive and/or (hypo)manic) within the year prior to baseline; and 4) a Young Mania Rating Scale (YMRS) [34] score of 12 or lower. The following exclusion criteria will be applied: 1) insufficient comprehension of the Dutch language; 2) previous participation in an eight-week mindfulness-based intervention; 3) having suffered a manic episode within three months before baseline; 4) a lifetime diagnosis of schizophrenia or schizoaffective disorder, current substance abuse disorder, organic brain syndrome, or antisocial or borderline personality disorder; 5) increased risk of suicide or aggression; 6) additional psychological interventions, such as CBT or trauma therapy, at the time of recruitment, baseline assessment or between T0 and T1; and 6) the presence of a concurrent significant medical condition impeding the ability to participate.

### Procedure

**Fig. 1:**
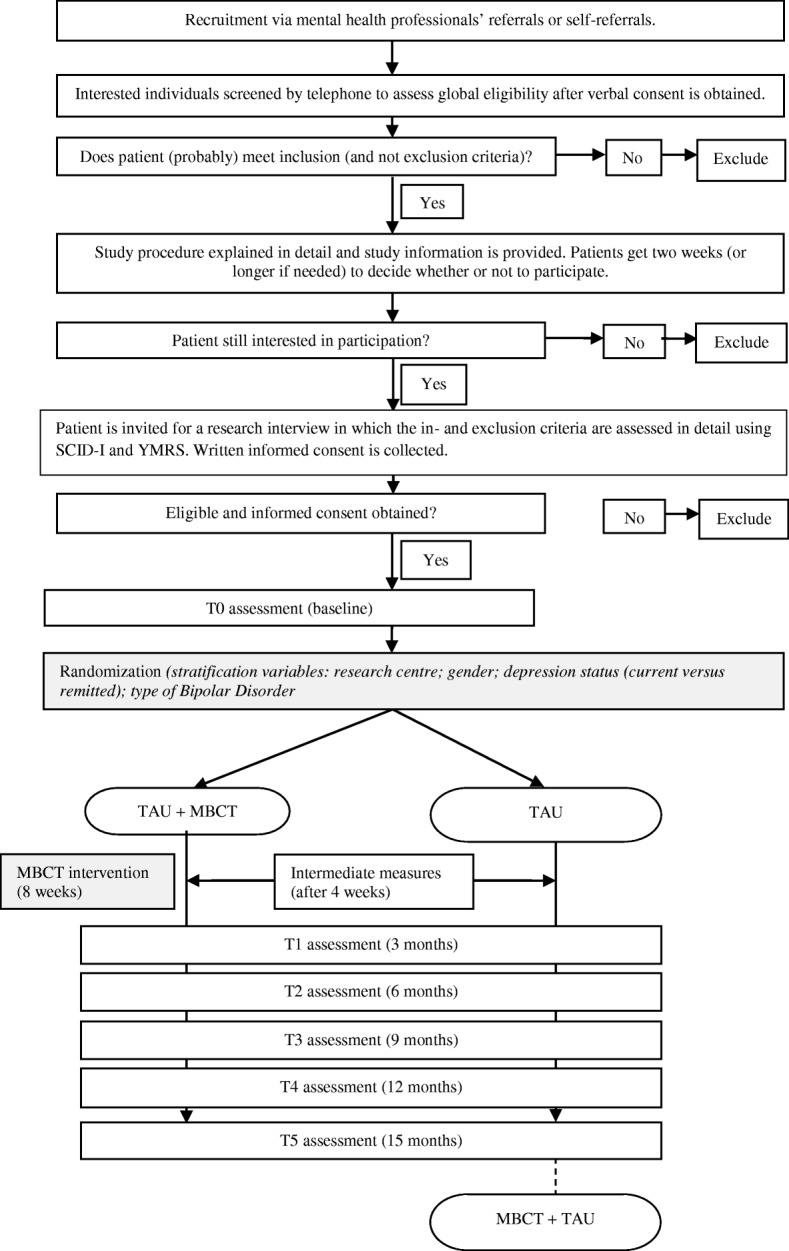
Flowchart of study procedures from referral to final assessment

Clinicians from the participating outpatient clinics will provide the first screening by selecting patients from their caseload who will most likely meet the in- and exclusion criteria of this study. To minimize the risk of “gatekeeping”, they will be asked to only exclude patients not meeting in- or exclusion criteria and to provide the reasons for exclusion on a form. Subsequently, potentially eligible participants will receive an invitation letter and information leaflet from their attending clinicians, after which they can contact the research team. Participants will be recruited by self-selection as well, for example by media advertisements by the Dutch patient association of adults with BD (Vereniging voor Manisch Depressieven en Betrokkenen). After verbal consent is obtained, participants will be invited for a screening by telephone to assess eligibility. Eligible participants will be invited for a research interview with a trained research assistant, where written informed consent will be obtained. During the research interview, participants will be thoroughly screened for in- and exclusion criteria with use of the SCID-I and YMRS. Subsequently, if participants are still eligible to participate, they will be invited for the baseline assessment, which consists of several clinician administered measures and self-report questionnaires (see Table 2). Blinded assessments of clinician-rated measurements will be obtained by trained research assistants at 3, 6, 9, 12, and 15 months follow-up. The 6, 9, and 12 months follow-up assessments will be conducted by telephone. Self-report questionnaires will be obtained at baseline, four weeks after start of the MBCT intervention (mid-treatment) and at 3, 6, 9, 12, and 15 months follow-up. Figure 1 provides a flowchart of the study procedures from referral to final assessment.

### Randomization and blinding

Randomization will be computerized using an Electronic Data Capture (EDC) program (CASTOR: https://www.castoredc.com/). Randomization will be stratified for 1) setting; 2) gender; 3) depression status (current vs. remitted); and 4) bipolar type I or II. In order to ensure balanced groups, we will use block randomization with block sizes of either 2, 4, or 6. Research assistants on site will be blinded for allocation and participants will be asked not to talk about their group allocation during assessments.

### Treatment as usual

Usual care of people with BD typically consists of pharmacotherapy (usually provided and monitored by a psychiatrist), and psycho-education and self-management interventions, e.g. maintaining biopsychological rhythm, detection and management of early signs of mood dysregulation, and improving coping skills (usually provided and monitored by a psychiatric nurse) [19]. Because of the clinical representativeness, we will not restrict TAU in any way and, therefore, participants will be allowed to switch, taper or augment their medication. It is expected that a large majority of participants in the current study will receive some form of pharmacotherapy. A careful record of this will be kept in order to examine and control for possible differences between the two groups.

### Intervention

The MBCT program developed in the current study is an adaptation of the original MBCT intervention for recurrent depression by Segal, Williams, and Teasdale [20]. The MBCT program was adapted to the needs of people with BD in terms of tailoring psycho-educative elements to BD (e.g. adding information and exercises focusing on mania), implementing more movement exercises, introducing the three-minute breathing space earlier and more often in the program, adding a partner session (session 6), and repeatedly bringing focus to self-care. These adaptations were based on qualitative feedback of 15 individuals with BD who participated in two consecutive pilot groups of traditional MBCT at the Radboud University Medical Centre (Nijmegen, the Netherlands). These qualitative data, focusing on barriers and facilitators, were collected during two focus groups.

The group-based MBCT program consists of eight weekly 2.5 h sessions, a 6-h silent day between session six and seven, and home assignments to practice both formal (e.g. body scan, sitting meditation) and informal meditative exercises (e.g. mindful routine activity) about 45 min per day. All MBCT interventions will be conducted at the respective mental health sites, with each group consisting of 8–10 participants. Table 1 provides an overview of the MBCT program as applied in the current study.

**Table 1:** Overview of MBCT sessions

Theme of session	Mindfulness exercises	Didactic teaching	Homework assignments
1. Automatic pilot	• Raisin exercise • Body scan • Three-minute breathing space	• Bipolar disorder • Rationale for mindfulness	• Body scan • Mindful eating • Mindful routine activity • Three-minute breathing space
2. Dealing with barriers	• Awareness of surroundings • Body scan • Sitting meditation – focus on breath • Three-minute breathing space	• Relationship between thoughts and feelings	• Body scan • Three-minute breathing space • Mindful routine activity • Pleasant events calendar
3. Mindfulness of the breath	• Sitting meditation – focus on breath and body • Three-minute breathing space • Floor yoga	• Awareness of pleasant events • Automatic positive thoughts (mania) • Recognition and managing core symptoms of mania	• Sitting meditation • Floor yoga • Mindful routine activity • Unpleasant events calendar • Three-minute breathing space
4. Staying present	• Sitting meditation – focus on breath, body, sounds, and thoughts • Three-minute breathing space • Mindful walking	• Awareness of unpleasant events • Automatic negative thoughts • Recognition and managing core symptoms of depression	• Sitting meditation/ mindful walking/ floor yoga/ body scan • Three-minute breathing space (regular) • Three-minute breathing space (coping) • Stressful events calendar
5. Allowing and letting be	• Sitting meditation –focus on breath, body, sounds, and thoughts • Standing yoga • Three-minute breathing space	• Acceptance • Stress • Helping and non-helping thoughts	• Sitting meditation • Standing yoga • Three-minute breathing space (coping) • Communication events calendar
6. Mindful communication *(partner session)*	• Standing yoga • Three-minute breathing space	• Mindfulness and bipolar disorder • Communication – listening and speaking	• Sitting meditation/ mindful walking/ floor yoga/ body scan • Three-minute breathing space • Creating crisis intervention action plan
Silent day - 6 hours			
7. Taking care of yourself	• Sitting meditation – focus on breath, body, sounds, thoughts, emotions, and choiceless awareness	• Recognizing symptoms of relapse • Keeping in balance • Relapse prevention plan	• Sitting meditation/ mindful walking/ floor yoga/ body scan • Three-minute breathing space • Adjusting relapse prevention plan Self-evaluation
8. The rest of your life	• Three-minute breathing space • Body scan • Three-minute breathing space	• Preventing relapse • Maintaining practice • Reflection	

#### Mindfulness teachers

Each MBCT intervention will be taught by two teachers, of whom at least one will qualify the advanced criteria of the Association of Mindfulness Based Teachers in the Netherlands and Flanders, which are in concordance with the good practice guidelines of the UK Network for Mindfulness-Based Teachers [35]. These criteria include the following: 1) a minimum of 150 h of education in MBSR/MBCT background and theory, training in formal and informal meditative practices, psycho-education and inquiry, supervision and teaching a MBSR/MBCT including a reflection report; 2) relevant professional training; 3) minimum of three years of practicing meditation regularly and attending retreats; 4) having attended a MBSR/MBCT intervention as a participant; 5) continued training; and 6) giving a minimum of two MBCT/MBSR interventions per two years. All mindfulness teachers will receive additional training in the study protocol at the start of the trial. Supervision meetings will be organized repeatedly during the intervention phase of the trial. Teacher competency will be assessed by the Mindfulness-Based Interventions – Teacher Assessment Criteria (MBI-TAC) [36]. Videotapes of a random selection of sessions will be assessed by assessors who are familiar with the MBCT program, and are proficient mindfulness teachers themselves (level 5) who have received training in the use of these assessment criteria. An early study of the psychometric properties of the MBI-TAC suggests it has good reliability, face validity, and promising evidence of validity [37].

### Outcome measures

#### Primary outcome measures

Table 2 provides an overview of the assessments. The primary outcome measure will be the post-intervention (3 months follow-up) total score on the *Inventory of Depressive Symptomatology – Clinician administered* (IDS – C), a 30-item clinician administered scale to assess depressive symptom severity [38]. Items are scored on a 0–3 point scale, with total score ranges from 0 to 84 (where < 13 = not depressed, 14–25 = mildly depressed, 26–38 = moderately depressed, 39–48 = markedly depressed, and 49 > severely depressed). The psychometric properties of the IDS-C have been shown to be highly acceptable in samples of 544 outpatients with major depression and 402 outpatients with BD [39].

**Table 2:** Overview of assessments

		T0 (baseline)	Tmid (4 weeks)	T1 (3 months)	T2 (6 months)	T3 (9 months)	T4 (12 months)	T5 (15 months)
Primary outcome								
IDS-C	Depressive symptoms	x		x	x	x	x	x
Secondary outcomes								
YMRS	(Hypo)manic symptoms	x		x	x	x	x	x
SCID-I	Relapse	x		x	x	x	x	x
ASRM	(Hypo)manic symptoms		x					
QIDS-SR	Depressive symptoms		x					
STAI	Anxiety symptoms	x		x	x	x	x	x
FAST	Overall functioning	x		x	x	x	x	x
MHC-SF	Positive mental health	x	x	x	x	x	x	x
FFMQ	Mindfulness	x	x	x	x	x	x	x
SCS-SF	Self-compassion	x	x	x	x	x	x	x
RPA-NL	Responses to positive affect	x	x	x	x	x	x	x
RRS-EXT	Brooding	x	x	x	x	x	x	x
Cost-effectiveness								
EQ-5D-5L	Quality of life and quality adjusted life years	x		x	x	x	x	x
TiC-P	Costs associated with illness	x		x	x	x	x	x

#### Secondary outcome measures

The *Young Mania Rating Scale* (YMRS) [34] will be used to assess (hypo) manic symptom severity. The YMRS is an 11-item clinician administered scale, with a total score range of 0 to 60 (where ≤12 indicates remission, 13–19 = minimal symptoms, 20–25 = mildly manic, 26–37 = moderately manic, and 38–60 = severely manic). The psychometric properties of the YMRS have been shown to be adequate [34].

The *Structured Clinical Interview for DSM-IV Axis I Disorders* (SCID-I) [33] will be used to retrospectively assess the occurrence of depressive and/or (hypo)manic relapses in the past three months at each assessment. The psychometric properties of the Dutch translation of the SCID-I have been shown to be excellent [40].

The *Prospective Life Chart, self-report* [41] will be used for participants to daily document the course and severity of recurrent mood episodes in order to gain more fine-grained information about the severity of (hypo)manic and/or depressive symptoms over time. The psychometric properties of the Life Chart have been shown to be adequate [41].

The *Altman Self-Rating Mania Scale* (ASRM) [42] will be used to assess the presence and severity of (hypo)manic symptoms during the intermediate measurements (4 weeks after start MBCT). The ASRM is a 5-item self-report questionnaire with score ranges from 0 to 20 (where 6 ≥ indicates a high probability of a (hypo)manic condition). The psychometric properties of the ASRM have been shown to be good [42].

The *Quick Inventory of Depressive Symptomatology* (QIDS-SR) [43] will be used to assess depressive symptom severity during the intermediate measurements (4 weeks after start MBCT). The QIDS-SR is a 16-item self-report questionnaire with total score ranges from 0 to 27. The psychometric properties of the QIDS-SR have been shown to be adequate [43].

The *State/Trait Anxiety Inventory* (STAI) [44] will be used to assess severity of anxiety symptoms. The STAI is a 20-item, self-report measure with a total score range of 20 to 80. The psychometric properties of the Dutch translation of the STAI have been shown to be adequate [45].

The *Functioning Assessment Short Test* (FAST) [46] will be used to assess overall functioning. The FAST is a 24-item measure that assesses impairment or disability in six specific areas of functioning, including 1) autonomy; 2) occupational functioning; 3) cognitive functioning; 4) financial issues; 5) interpersonal relationships; and 6) leisure time. Psychometric properties of the FAST and its ability to detect differences between euthymic and acute bipolar patients have been shown to be excellent [46].

The *Mental Health Continuum – Short form* (MHC-SF) [47] will be used to assess emotional, psychological, and social well-being. The MHC-SF is a 14-item measure with a total score range of 0 to 70. The psychometric properties of the MHC-SF have been shown to be adequate [47].

The *brooding subscale* of the extended version of the *Ruminative Response Scale* (RRS-EXT) [48] will be used to assess levels of brooding, also known as rumination. The brooding subscale consists of five items and has been shown to have adequate psychometric properties [48].

The *Reponses to Positive Affect Questionnaire - Dutch Version* (RPA-NL) [9] will be used to assess responses to positive affective states. The RPA-NL is a 17-item self-report questionnaire consisting of three subscales, including: 1) dampening of positive affect; 2) self-focused positive rumination; and 3) emotion-focused positive rumination. The psychometric properties of the English [9] and Dutch [10] version of the RPA have been shown to be adequate.

The *Five Facet Mindfulness Questionnaire - Short Form* (FFMQ-SF) [49], a 24-item questionnaire, will be used to assess different aspects of mindfulness, including: 1) observing, 2) describing, 3) acting with awareness, 4) non-judging of inner experiences, and 5) non-reactivity to inner experiences. The psychometric properties of the FFMQ-SF have been shown to be adequate [49].

The *Self Compassion Scale – Short form* (SCS-SF) [50] will be used to assess levels of self-compassion. The SCS-SF is a 12-item measure that consists of three concepts that are related to self-compassion, including 1) self-kindness versus self-judgment; 2) common humanity versus isolation; and 3) mindfulness versus over-identification. The psychometric properties of the SCS-SF have been shown to be adequate [50].

#### Cost-effectiveness

The *EQ-5D-5 L* [51] will be used to measure the Quality of Life and the Quality Adjusted Life Years. The EQ-5D-5 L consists of five dimensions, including: 1) mobility; 2) self-care; 3) usual activities; 4) pain/discomfort; and 5) anxiety/depression. The psychometric properties of the EQ-5D-5 L have been shown to be adequate [52].

The *Trimbos/iMTA questionnaire for costs associated with psychiatric illness* (TiC-P) [53] will be used to assess recourse use, such as use of care, medication, and illness related to work. It measures both direct costs, i.e. care consumption of people suffering from psychiatric illness, and indirect costs, i.e. costs associated with production loss. The psychometric properties of the TiC-P have been shown to be adequate [54].

### Adherence

Participants in the MBCT + TAU condition will be asked to daily document adherence to mindfulness practice. For this purpose, a calendar was designed on which participants will be asked to fill out the number of minutes daily spent on formal and informal mindfulness practices during the intervention. During follow-up, adherence will be retrospectively determined using a short questionnaire, asking which mindfulness exercises they still practice, how often and for how long. Participants in both conditions will be asked to document their medication adherence as well, using the *Prospective Life Chart, self-report* [41].

### Safety monitoring

In accordance with Good Clinical Practice guidelines, (serious) adverse events, both related and unrelated to the study, will be reported. During follow-up assessments, participants will be explicitly asked whether they have experienced any undesirable (medical) incidents during the study period. The occurrence, intensity and duration of these (serious) adverse events will be documented carefully. Furthermore, participants in the MBCT + TAU condition will be asked to daily document any (serious) adverse reactions ((S)AR) they might experience in response to mindfulness practice during the MBCT intervention. For this purpose, a log was designed on which twelve of the most likely (S)AR related to mindfulness practice for bipolar patients are documented. These (S)AR are inspired by earlier studies on adverse effects in meditation [55–57] and include the following: 1) re-experiencing of traumatic memories; 2) overwhelming or uncontrollable feelings of depression; 3) uncontrollable feelings of happiness or grandiosity; 4) uncontrollable feelings of irritability; 5) sudden (increase) of anxiety or panic; 6) feelings of derealization; 7) feelings of depersonalization; 8) feelings of distrust to others; 9) feelings of doubt towards the self; 10) visual hallucinations; 11) auditory hallucinations; and 12) unusual physical sensations. Participants will be asked to indicate how often they experienced certain (S)AR, whether they consider these reactions to be causally related to meditation, the intensity of these (S)AR in a scale from 1 to 10 (were 1 = low intensity and 10 = high intensity), and how they reacted in response to this (S)AR. In the long-term, if not reported spontaneously, (S)AR will be retrospectively determined during qualitative interviews.

### Statistical analysis

#### Sample size calculation

The sample size calculation was based on the estimated change in depressive symptom severity from pre- to post-intervention, as reported in a similar study about the efficacy of MBCT in recurrent depression conducted at the Radboud University Medical Centre [24]. This study found an effect size of 0.5 for reduction of depressive symptoms in people with recurrent (unipolar) depression, who were partly in remission (*n* = 124), or partly currently depressed (*n* = 58). Based on a two-sided test with an alpha of .05 and a power of 80%, with an estimated effect size of 0.5, including a design-factor of 1 – r^2^ (0.75), and taking account of a conservative estimate of 40% loss to follow-up, the current study will intend to recruit 160 patients (intervention group *n* = 80, control group *n* = 80).

#### Statistical analysis

All data will be analyzed and reported according to the CONSORT guidelines [58], using intention-to-treat and per-protocol analyses. Inadvertently unequally distributed baseline parameters between the conditions will be included as covariates. Assumptions of normality will be checked and, in case of lack of normality, the bootstrapping procedure will be used to account for this problem [59]. Sensitivity analyses will be conducted with different scenarios of imputed data sets to examine the influence of missing data on the pattern of outcomes.

Primary analysis will be aimed at comparing depressive symptom severity at three months after baseline between MBCT + TAU and TAU. In order to investigate consolidation of treatment effect, outcomes will be assessed at 6, 9, 12, and 15 months follow-up. Secondary analyses will be aimed at comparing the treatment effects of MBCT + TAU versus TAU on (hypo)manic and anxiety symptom severity, relapse rates, and overall functioning and positive mental health, post-intervention and up to 15 months follow-up. Multilevel analysis will be used to account for the cluster-randomized design and, therefore, the hierarchical structure of the data, with outcome variables at pre- and postmeasurements at the lowest level, nested within individuals, nested within treatment groups (in the intervention condition), and nested within condition (MBCT + TAU versus TAU). Mediation analysis will be conducted to investigate whether possible clinical effects of MBCT in primary outcome measures are mediated by rumination on positive and negative affect, mindfulness skills, and self-compassion [60]. Additional analyses will be performed within subgroups who suffered < 12 or ≥ 12 mood episodes, and within subgroups with and without a current depressive episode.

The economic evaluation will be based on the general principles of a cost-utility, cost-effectiveness, and budget-impact analysis, comparing MBCT + TAU versus TAU. Primary outcomes of the economic evaluation will be quality adjusted life years (QALYs), treatment response (scores on IDS-C), and both direct and indirect costs [61]. The incremental cost-effectiveness ratio (ICER) will be computed using non-parametric bootstrap methods to account for skewness of the cost-data. The cost-effectiveness ratio will be stated in terms of costs per outcome rate (IDS-C), while the cost-utility ratio will focus on the cost per QALY gained. The budget impact analysis (BIA) will be used as outlined by the IPSOR Task Group [62, 63] to assess how health care budgets change when MBCT is offered over a range of implementation levels. The BIA will be conducted from various perspectives, including: 1) the societal perspective (i.e. productivity loss); 2) the perspective of the public purse; and 3) the perspective of the health care insurer. In each perspective different scenarios will be assessed, in which the intervention is offered to 40, 60, and 80% of the target group, and an extreme scenario in which 100% of the target group will be receiving MBCT interventions. These scenarios will be compared with a base-case scenario where 0% of the target group is offered MBCT (reflecting TAU). The BIA will be based on a health-economic simulation excel model, based on modeling techniques outlined by Briggs, Claxton, and Sculpher [64], and following IPSOR modeling guidelines [65]. The BIA will be conducted according to the Dutch guidelines [66], taking into account the complexity and dynamics of clinical practice and specific characteristics of the Dutch health care system.

## Discussion

The current trial will be the first properly powered, multicenter, randomized controlled trial of MBCT for BD in the Netherlands and the second world-wide. It will provide high-level evidence of the relative long-term effectiveness of MBCT + TAU compared to TAU alone by including follow-up measurements up to 15 months en keeping close track of treatment adherence and occurrence of (serious) adverse reactions. The study will be conducted in five specialized clinics for bipolar disorders in order to enhance generalizability. From a societal perspective, the current trial will assess whether adding MBCT to TAU is cost-effective. This study will provide valuable insight into the accessibility and (cost-) effectiveness of MBCT in terms of clinician-rated and self-rated symptoms of BD, relapse rates, positive mental health, and overall functioning. Furthermore, this study might elucidate in which stage(s) of the illness MBCT might be helpful in relieving residual mood symptoms.

## Abbreviations

(S)AR: (serious) adverse reaction
BIA: Budget impact analysis
CBT: Cognitive behavioral therapy
CHIME: Comprehensive inventory of mindfulness experience
DSM-5: Diagnostic and statistical manual of mental disorders – 5th edition
FAST: Functioning Assessment Short Test
ICER: Incremental cost-effectiveness ratio
MBCT: Mindfulness-Based Cognitive Therapy
MBI-TAC: Mindfulness-Based Interventions – Teacher Assessment Criteria
MHC-SF: Mental Health Continuum- Short Form
OASIS: Overall anxiety severity and impairment scale
QALY: Quality adjusted life years
SCID-I: Structured Clinical Interview for DSM-IV Axis I Disorders
SCS-SF: Self-Compassion Scale – Short Form
STAI: State/Trait Anxiety Inventory
TAU: Treatment as usual
TiC-P: Trimbos/iMTA questionnaire for costs associated with psychiatric illness
YMRS: Young Mania Rating Scale
